# A comparative study of fatigue and processing speed in patients with multiple sclerosis treated with natalizumab or rituximab

**DOI:** 10.1177/20552173241252566

**Published:** 2024-05-26

**Authors:** Johan Hellgren, Maria Compagno Strandberg, Kristina Källén, Anders Svenningsson

**Affiliations:** Clinical Sciences Helsingborg Unit, Department of Clinical Sciences Lund, Lund University, Lund, Sweden; Neurology Section, Department of Specialised Medicine, 59579 Helsingborg General Hospital, Helsingborg, Sweden; 403181 Neurology Lund Unit, Department of Clinical Sciences Lund, 156327 Lund University, Lund, Sweden; Department of Neurology, Rehabilitation medicine, Memory disorders and Geriatrics, Skånes University Hospital, Lund, Sweden; Clinical Sciences Helsingborg Unit, Department of Clinical Sciences Lund, Lund University, Lund, Sweden; Neurology Section, Department of Specialised Medicine, 59579 Helsingborg General Hospital, Helsingborg, Sweden; Department of Neurology, Rehabilitation medicine, Memory disorders and Geriatrics, Skånes University Hospital, Lund, Sweden; Department of Clinical Sciences, 411426 Karolinska Institutet, 72227 Danderyd Hospital, Stockholm, Sweden; Department of Neurology, Danderyd Hospital AB, Stockholm, Sweden

**Keywords:** Multiple sclerosis, rituximab, natalizumab, fatigue, cognition, patient reported outcome measures

## Abstract

**Background:**

Fatigue is the most debilitating symptom in patients with multiple sclerosis (MS). Natalizumab and rituximab are the most used MS disease modifying therapies in Sweden, but comparative data on the effect on fatigue is sparse.

**Objective:**

Primary objective was to compare fatigue levels between patients on natalizumab and rituximab. As secondary objective, we assessed processing speed, an attention domain quality, between treatment groups.

**Method:**

In this Swedish multicentre cross-sectional study, patients with relapsing-remitting MS and >24 months treatment duration were identified in the Swedish MS-registry. Fatigue was assessed using the Fatigue Scale for Motor and Cognitive functions (FSMC) and processing speed using Symbol Digit Modalities Test (SDMT).

**Results:**

128 patients were enrolled (natalizumab: 56, rituximab: 72). No significant differences in FSMC were found when adjusting for potential confounders (p = 0.936), with age having the biggest impact, correlating with increased fatigue. Individuals on natalizumab performed significantly better on SDMT at cross-section (natalizumab 64.7, rituximab 56.2; p = 0.003), with an improvement from treatment initiation, compared to rituximab (change: natalizumab 8.9, rituximab −1.0; p = 0.002).

**Conclusion:**

We found no difference in fatigue levels between natalizumab and rituximab cohorts. Patients treated with natalizumab showed significantly better results on SDMT than patients on rituximab.

## Introduction

Over 80% of patients with multiple sclerosis (MS) have been reported to suffer from fatigue.^1^ It often presents at disease onset, might precede other clinical symptoms, and persists throughout the course of the disease.^2,3^ An expert panel summarised the main clinical features of fatigue: “A subjective lack of physical and/or mental energy that is perceived by the individual or caregiver to interfere with usual and desired activities”.^4^ Both self-assessed fatigue and quantified cognitive fatigability were reported as the main reasons for reduced work capability and strongly associated with decreased working hours and disability pension.^5,6^ Assessing the impact of fatigue at the individual level presents a challenge due to its highly subjective nature and susceptibility to multiple confounding factors such as coexisting depression and anxiety.^7^

Natalizumab (NTZ) and rituximab (RTX) are currently the most used disease-modifying therapies (DMTs) in Sweden and account for 67% (NTZ 10%, RTX 57%) of all prescribed MS DMTs. These monoclonal antibody treatments are both considered as first- and second-line therapy in relapsing-remitting MS (RRMS). Several studies have proven the safety and efficacy of these DMTs as they almost eliminate clinical relapses and new lesion formation on MRI.^8–10^ However, comparative data on the effect on fatigue are scarce.

Frequent clinical observations from included study centres suggested that individuals treated with NTZ suffer less from fatigue than those on RTX. This study aimed to investigate differences between patients treated for at least 24 months with NTZ or RTX on patient-reported outcome measures (PROMs), with a focus on fatigue as a primary outcome, and on cognitive performance in the attention domain as a secondary outcome.

## Methods and materials

### Study design

This was a comparative Swedish multicentre cross-sectional study, comparing NTZ and RTX regarding PROMs and the symbol digit modalities test (SDMT). The other parts of the comprehensive study protocol (spinal fluid, blood analyses and 7-tesla MRI imaging) will be reported in separate publications.

Participants were recruited from three out-patient neurology clinics with the majority from Skåne County in southern Sweden. Centres included were Helsingborg General Hospital (secondary referral centre), Skåne University Hospital (tertiary referral centre), and a few participants from Danderyd Hospital in Stockholm County (secondary referral centre).

Patients registered in the Swedish MS registry (SMSreg; https://www.neuroreg.se/multipel-skleros/) were eligible. Inclusion criteria were age 18–60 years, a diagnosis of RRMS according to the McDonald criteria,^11^ and treatment with either NTZ or RTX for at least 24 months before inclusion. Exclusion criteria were prior treatment with alemtuzumab or haematopoietic stem cell transplantation, pregnancy, breastfeeding, comorbidity with other neurodegenerative diseases, malignancies, and MRI-findings inconsistent with MS. Participants were either contacted by a research nurse when they went to the clinic for treatment, via letter, or via an online platform for medical information and communication between patients and health care providers in Sweden, called 1177. Alternatively, a research nurse or researcher (JH), contacted patients by phone. Inclusion and data collection took place between November 2020 and January 2024, also constituting the cross-section period.

### Data collection

Demographic data was extracted from the SMSreg. As part of annual follow-up outlined by the Swedish MS Association, patients perform SDMT and fill out a set of nationally consensus-based PROMs, which are entered into the SMSreg (www.mssallskapet.se). In case data collection was done >12 months before the time of cross-section participants were contacted to update their PROMs. FSMC is not included in the yearly set of consensus-based PROMs and was added to the study protocol at the time of cross-section. Data on clinical features was primarily obtained from medical records.

### Ethical considerations

The Swedish Ethical Review Authority granted permission for the study (DNR: 2020-03608, 2020-06639, 2021-05389). Access to the SMSreg was in accordance with registry regulations and granted by registry holders. All participants provided written informed consent before enrolment.

### Assessment of fatigue, health-related quality of life and clinical features

#### Fatigue scale for motor and cognitive functions (FSMC)

FSMC is a 20-item questionnaire assessing fatigue in relation to everyday life activities, validated for diagnosing and assessing fatigue in MS.^12^ Ten items concern cognitive and motor fatigue, respectively. A Likert scale ranging from 1 (“completely disagree”) to 5 (“completely agree”) rates each item expressed as a statement. Sum scores graded the level of fatigue according to predetermined threshold values for total, as well as cognitive and motor fatigue separately. High scores indicate worse fatigue.

#### Multiple sclerosis impact scale −29 items (MSIS-29) 

MSIS-29 is a self-reported 29-item questionnaire measuring the physical and psychological impact of MS in daily life.^13^ Nine items concern psychological and 20 physical impairments. Each item, presented as a question, addresses difficulties due to MS during the last two weeks and is graded on a Likert scale ranging from 1 (“not at all”) to 5 (“extremely much”). Summary scores for each category were calculated and transformed into a value ranging from 0–100, with higher value indicating poorer health-related quality of life.

#### Treatment satisfaction questionnaire for medications (TSQM-10)

TSQM-10, a modified version of TSQM-9, is a ten-item questionnaire for assessment of treatment satisfaction^14^ with an additional question about side effects as applied in the SMSreg.^15^ Each item, presented as a question, is graded on a Likert scale. The questions address: 1) treatment effectiveness, drug administration and side effects (item 1–7, graded 1–7), 2) belief in benefits of treatment (items 8–9, graded 1–5), and 3) overall satisfaction (item 10, graded 1–7). A maximum of 59 points summed up items 1–9, while item 10 was presented separately. A high score corresponds to high treatment satisfaction.

#### Symbol digit modalities test (SDMT)

The substitution test SDMT measures information processing speed, a cognitive quality in the attention domain.^16^ A form with 110 symbols in a random sequence is presented to participants. Each symbol should be paired with a pre-specified number between 1 and 9. Participants achieve a score based on the number of successful pairings in 90 s. A difference of 8 points or more was deemed clinically significant.^17^

#### Employment and education status

Participants were asked by a research nurse if they currently were: 1) working/studying and, if so, to what degree (fulltime or part time), or unemployed, or neither; 2) on sick leave and, if so, to what degree (25, 50, 75 or 100%); 3) on disability pension and, if so, to what degree (25, 50, 75 or 100%); and 4) on parental leave and, if so, to what degree (free text answer).

The online platform 1177 was used for assessment of educational level. Participants were asked to check their highest level of education given three choices: 1) Compulsory School, 2) Upper Secondary School or 3) Post-secondary School.

#### Clinical features

Information on total burden of white matter lesions on MRI, annual relapse rate, comorbidity, adverse events, and concurrent medical treatment, were retrieved from medical records. The total lesion burden on T_2_-weighted images was categorised into 1–9, 10–20, and >20, in accordance with Swedish guidelines of MRI in treatment and follow-up of MS.^18^

#### EDSS

The SMSreg provided information on EDSS. In case of missing data, medical records provided information on neurological examination based on EDSS at annual follow-ups. In cases without assigned EDSS, a consensus discussion within the research team provided an EDSS estimate based on previous EDSS and examination findings. If the research team was unable to estimate the EDSS using SMSreg and medical record review, the EDSS was administered by telephone, either by a research nurse or a researcher.^19^

### Statistical analysis

Lack of previous published data of fatigue in RTX for MS made a power estimate difficult. Based on clinical experience, we hypothesised that there would be a difference in FSMC score between treatment groups of approximately 15%, in NTZs favour.^20^ For a 90% power the sample size estimate was 50 participants in each group with the assumption that the standard deviation (SD) was close to 1.0 and p was set to 0.05. Statistical significance was set at p < 0.05.

Linear regression models compared continual outcomes between groups (i.e., MSIS-29, FSMC, SDMT). A logistic regression model compared the differences between groups when FSMC had a binary outcome with an estimated clinically significant cut-off between mild and moderate fatigue. A forest plot showed the potential impact of confounders on results. In the linear regression models, potential confounding factors were EDSS, age, number of MRI-lesions at treatment initiation, disease duration, sex, and time on RTX and NTZ. Educational level was included in the regression models of SDMT. The logistic regression models limited the potential confounders to sex, age, and time on RTX and NTZ due to the limited sample size.

In a sensitivity analysis, participants in the RTX group that were initially treated with NTZ and then switched to RTX on the indication of high John Cunningham Virus index, were excluded. This was to eliminate the possible negative bias introduced in the RTX group from an involuntary change of an effective and well-functioning medication.

## Results

### Inclusion and characteristics

**Figure 1.**
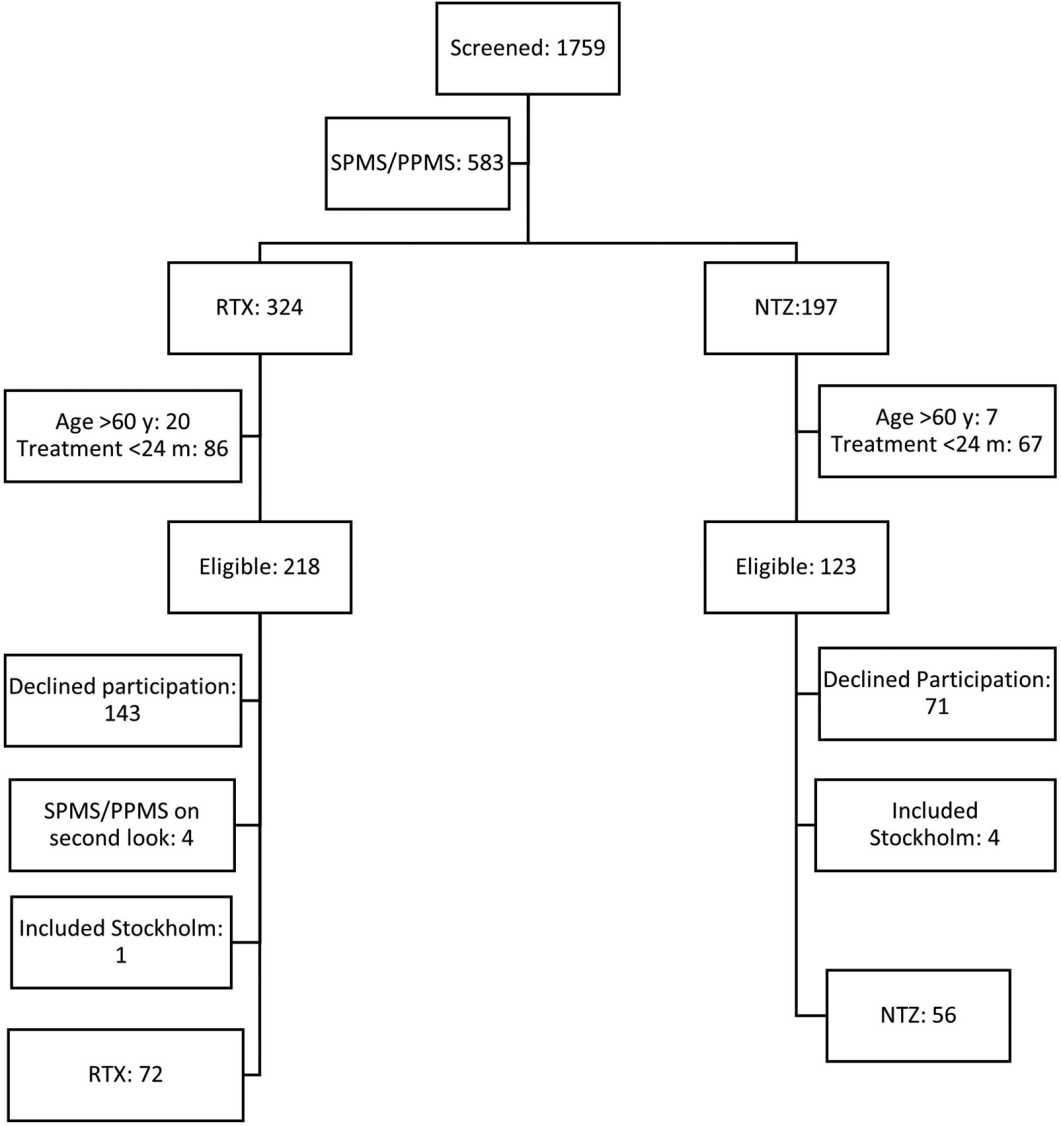
Depicts the inclusion process.

Abbreviations: SPMS, secondary progressive multiple sclerosis; PPMS, primary progressive multiple sclerosis; NTZ, natalizumab; RTX, Rituximab.

Figure 1 shows the inclusion process. We screened 1759 patients with MS at Skåne University Hospital and Helsingborg General Hospital, of which 341 met inclusion criteria. Five patients were consecutively enrolled from the clinical practice at the MS team at Danderyd Hospital in Stockholm to achieve the pre-estimated power calculation number for the primary variable FSMC. Individuals who declined participation stated lumbar puncture and the long scanning time association with the 7T-MRI as the main reasons.

Table 1 shows patients’ characteristics, total lesion burden at treatment initiation, annual relapse rate from treatment initiation to cross-section, number of participants on fatigue medication and antidepressant medication, educational level, and employment status at the time of cross-section. Participants treated with RTX were on average older with a longer disease duration. A larger proportion of the participants in the NTZ cohort worked full-time (NTZ n = 31, 55.4%; RTX n = 32, 44.4%) and were to a lesser extent on sick leave or received disability pension at cross-section compared to the RTX cohort. Annual relapse rate was very low in both treatment groups (NTZ = 0.024, RTX = 0.014). During the year prior to cross-section, no participants showed signs of disease activity (relapses, new MRI-lesions, disease progression).

**Table 1.** Patients demographic and disease specific characteristics, relevant comorbidities and disease related socioeconomic information at cross-section.

Table 1: Demographics and characteristics			
	RTX (n = 72)	NTZ (n = 56)	Total (n = 128)
**Age, mean years (SD)**	45.6 (8.6)	40 (8.8)	43.2 (9.1)
**Disease duration, median years (range)**	12.6 (2.3–31.0)	10.8 (2.8–42.1)	11.6 (2.3–42.1)
**Treatment time, median years (range)**	4.0 (2.2–9.4)	6.4 (2.5–14.8)	4.5 (2.2–14.8)
**EDSS, median (range)** *Missing: n = 1*	1.5 (0–6.0)	1.0 (0–6.5)	1.0 (0–6.5)
**Female, n (%)**	55 (76)	49 (88)	104 (81)
**Comorbidity, n (%)**	5 (6.9)	2 (3.5)	7 (5.5)
Crohns disease	1 (1.4)	0	1 (0.8)
Endometriosis	1 (1.4)	2 (3.5)	3 (2.3)
Sjogrens Syndrome	1 (1.4)	0	1 (0.8)
Sarcoidosis	1 (1.4)	0	1 (0.8)
Rheumatoid Arthritis	1 (1.4)	0	1 (0.8)
**MRI brain lesions at treatment initiation, n (%)**			
1–9	20 (27.8)	9 (16.1)	29 (22.6)
10–20	17 (23.6)	16 (28.6)	33 (25.8)
> 20	35 (48.6)	31 (55.4)	66 (51.6)
Annual Relapse Rate*	0.014	0.024	0.02
**Antidepressant medication, n (%)**	15 (21)	6 (11)	21 (16)
**Fatigue medication, n (%)** *Missing: RTX n = 1, NTZ n = 4*	11 (15.3)	4 (7.1)	15 (11.7)
**Education, n (%)** *Missing: RTX, n = 2, NTZ, n = 4*			
Compulsory School	3 (4)	1 (2)	4 (3)
Upper Secondary School	18 (25)	7 (13)	25 (20)
Post-secondary Education	49 (68)	44 (79)	93 (73)
**Occupation and sick leave, n (%)** *Missing: RTX n = 2, NTZ n = 1*			
No sickness benefit	37 (51.4)	38 (67.9)	75 (58.6)
Disability pension 100%	8 (11.1)	3 (5.6)	11 (8.6)
Disability pension <100%	12 (16.7)	8 (14.3)	20 (16.7)
Sick leave	13 (18.1)	6 (10.1)	19 (16.8)

* Annual relapse rate is presented from treatment initiation to cross-section.

Abbreviations: EDSS, Expanded Disability Status Scale; RTX, rituximab; NTZ, natalizumab; SD, standard deviations; MRI, magnetic resonance imaging.

### Patient reported outcome measures

Table 2 summarises PROMs and SDMT results. There was no significant difference between groups when comparing mean FSMC ordinal values. FSMC was thereafter dichotomised into two categories: 1) no and mild fatigue or 2) moderate and severe fatigue. There was a significantly higher risk for patients treated with RTX, compared with NTZ, for a higher crude value of all FSMC subscales. None of these results were significant after adjustment for potential confounders.

**Table 2.** PROMs and SDMT results on group level at cross-section and SDMT change from treatment initiation. No statistical analysis was performed on TSQ10, the tenth question covering overall treatment satisfaction.

Table 2: PROMs and SDMT				
	RTX	NTZ	P-value (crude)*	P-value (adj.)*
**FSMC, mean (SD)**				
Cognitive	29.2 (13.2)	25.5 (11.6)	0.123	0.985
Motor	28.2 (13.3)	23.6 (11.4)	0.058	0.857
Total	57.4 (26.3)	49.1 (22.3)	0.080	0.936
**MSIS-29, median (range)**				
Physical	19 (0–94)	11 (0–83)	0.032	0.149
Psychological	28 (0–91)	16 (0–86)	0.09	0.563
**SDMT, mean (SD)**				
Cross-section	56.2 (12.0)	64.7 (12.8)	<0.001	0.003
Treatment initiation	56.6 (11.3)	55.8 (12.1)		
Change^ꝉ^, mean (95% CI)	−1.0 (−2.7–0.8)	8.9 (6.0–11.8)	<0.001	0.002
**TSQ10, median (range)**	6 (4–7)	6 (4–7)	N/A	N/A

* Crude p-values represent the differences between treatment groups at cross-section prior to adjustment for potential confounders. Adjusted p-values represent the difference after adjustment. Statistical significance was set to *p* < 0.05.

^ꝉ^ The mean of calculated SDMT-change at group level, from treatment initiation to cross-section.

Abbreviations: FSMC, The Fatigue Scale for Motor and Cognition Functions; MSIS-29, Multiple Sclerosis Impact Scale; SDMT, Symbol Digit Modalities Test; TSQ10, Treatment Satisfaction Questionnaire question 10; RTX, Rituximab; NTZ, Natalizumab; adj., adjusted.

There were no significant differences when comparing mean MSIS-29 physical and psychological subscales between the two treatment groups after adjustments for confounders.

Overall treatment satisfaction by TSQM was high. Most participants in both groups were very or extremely satisfied with their DMTs (score 6 or 7; NTZ: n = 66, 92%; RTX: n = 43, 79%) and no participant scored below “somewhat satisfied” (score 4).

**Figure 2.**
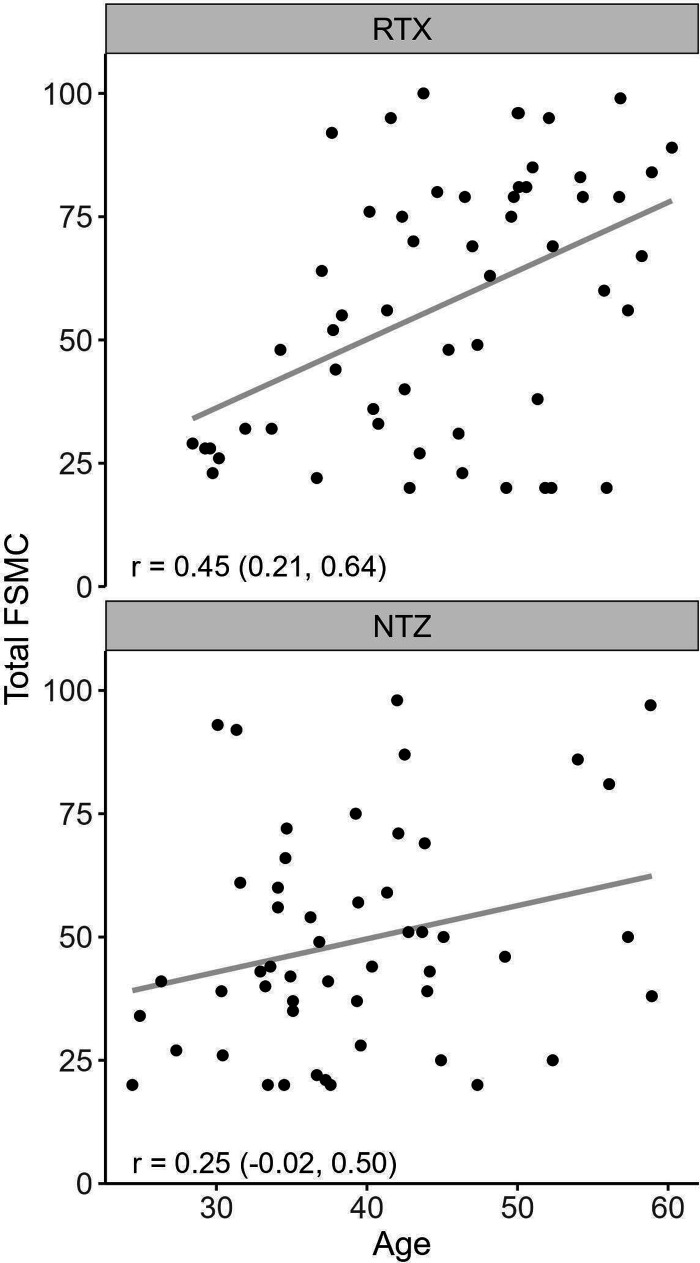
Depicts the relationship between age and Total FSMC for each treatment cohort, r = correlation coefficient with 95% confidence interval. Upper panel is the RTX and lower panel the NTZ cohort.

Abbreviations: FSMC, Fatigue Scale for Motor and Cognitive Functions; RTX, rituximab; NTZ, natalizumab.

**Figure 3.**
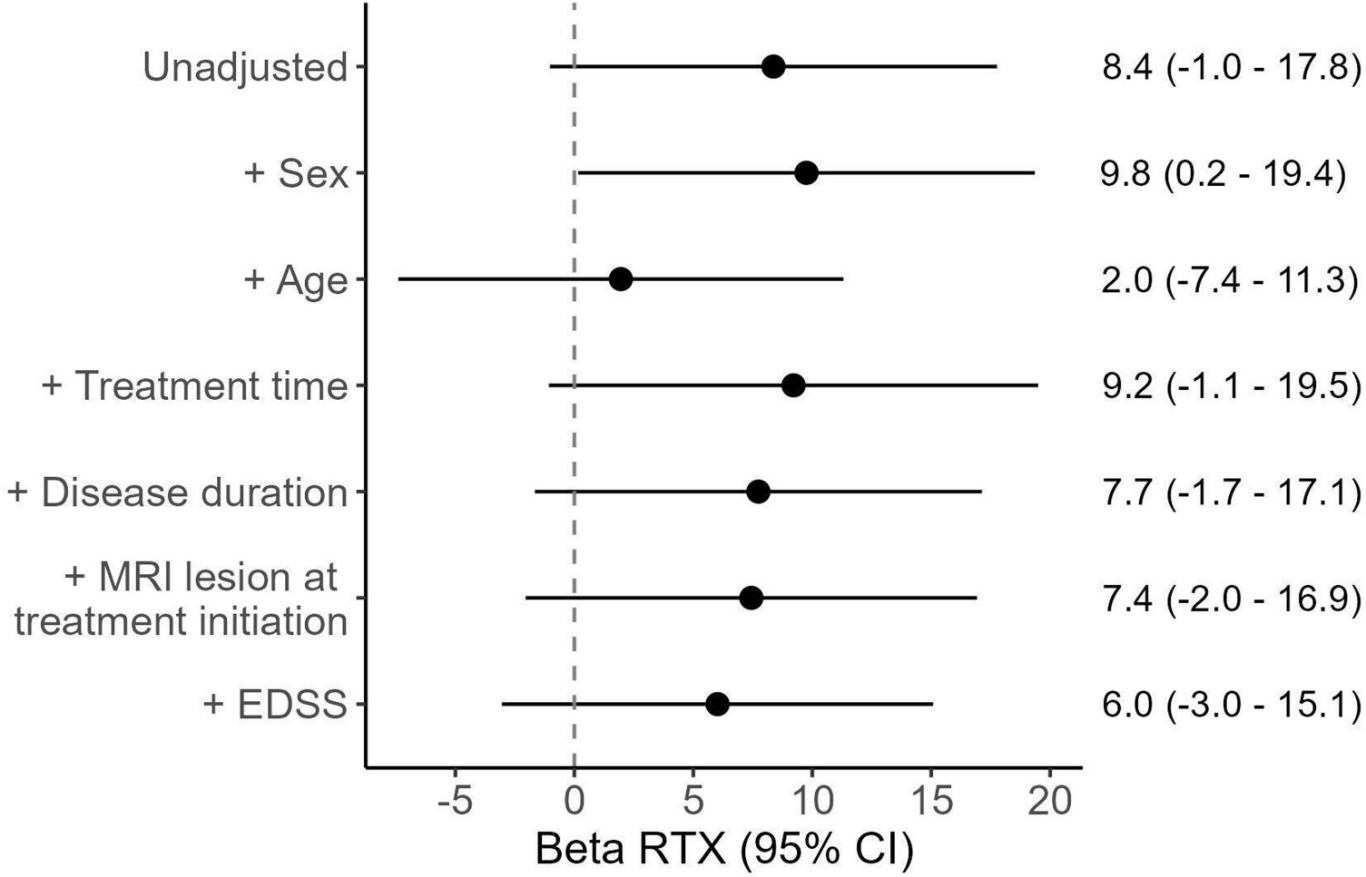
Displays the beta coefficient for RTX vs NTZ in the unadjusted model as well as several adjusted models, each being adjusted by a single confounder, to illustrate each confounders’ impact on the association between RTX and FSMC total. The Y-axis shows potential confounding factors and the X-axis shows the effect of RTX when modified for each factor, with age having the biggest impact on the beta value.

Abbreviations: RTX, rituximab; FSMC, Fatigue Scale for Motor and Cognitive Functions; EDSS, Expanded Disability Status Scale; MRI, Magnetic resonance imaging.

Figures 2 and 3 display the importance of age in fatigue evaluation of RRMS. Figure 3 is an explorative forest plot of the effect of potential confounders on FSMC total score. It shows that age had the largest impact on fatigue severity compared to other factors.

### Symbol digit modalities test

Participants in the NTZ cohort performed significantly better on SDMT compared to the RTX cohort. This result remained significant after adjusting for sex, age, disease duration, education, treatment duration and number of MRI lesions at treatment initiation. Furthermore, statistical significance remained unchanged in the sensitivity analysis excluding individuals in the RTX cohort previously treated with NTZ (p = 0.012).

Mean SDMT difference from treatment initiation to cross-section showed a significant improvement in the NTZ cohort not seen in the RTX group. The results remained significant after adjustment for potential confounders. However, the sensitivity analysis showed no significant difference (p = 0.057).

**Figure 4.**
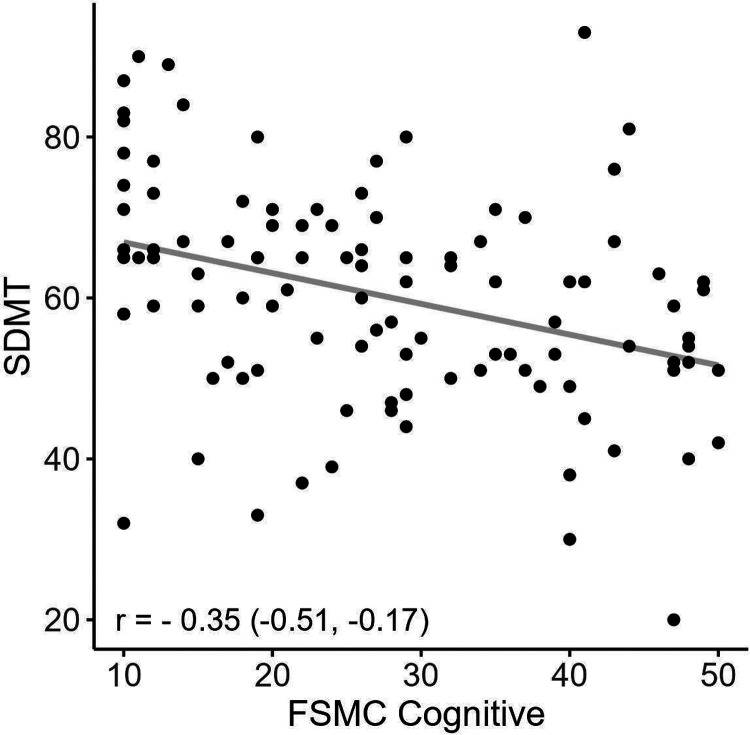
Displays the significant correlation between SDMT and the FSMC cognitive subscales, r = correlation coefficient with 95% confidence interval.

Abbreviations: SDMT, Symbol Digit Modalities Test; FSMC; The Fatigue Scale for Motor and Cognitive Functions.

Figure 4 displays the correlation between SDMT and the cognitive subscale of FSMC. Increasing cognitive fatigue significantly correlated with decreasing SDMT results.

**Figure 5.**
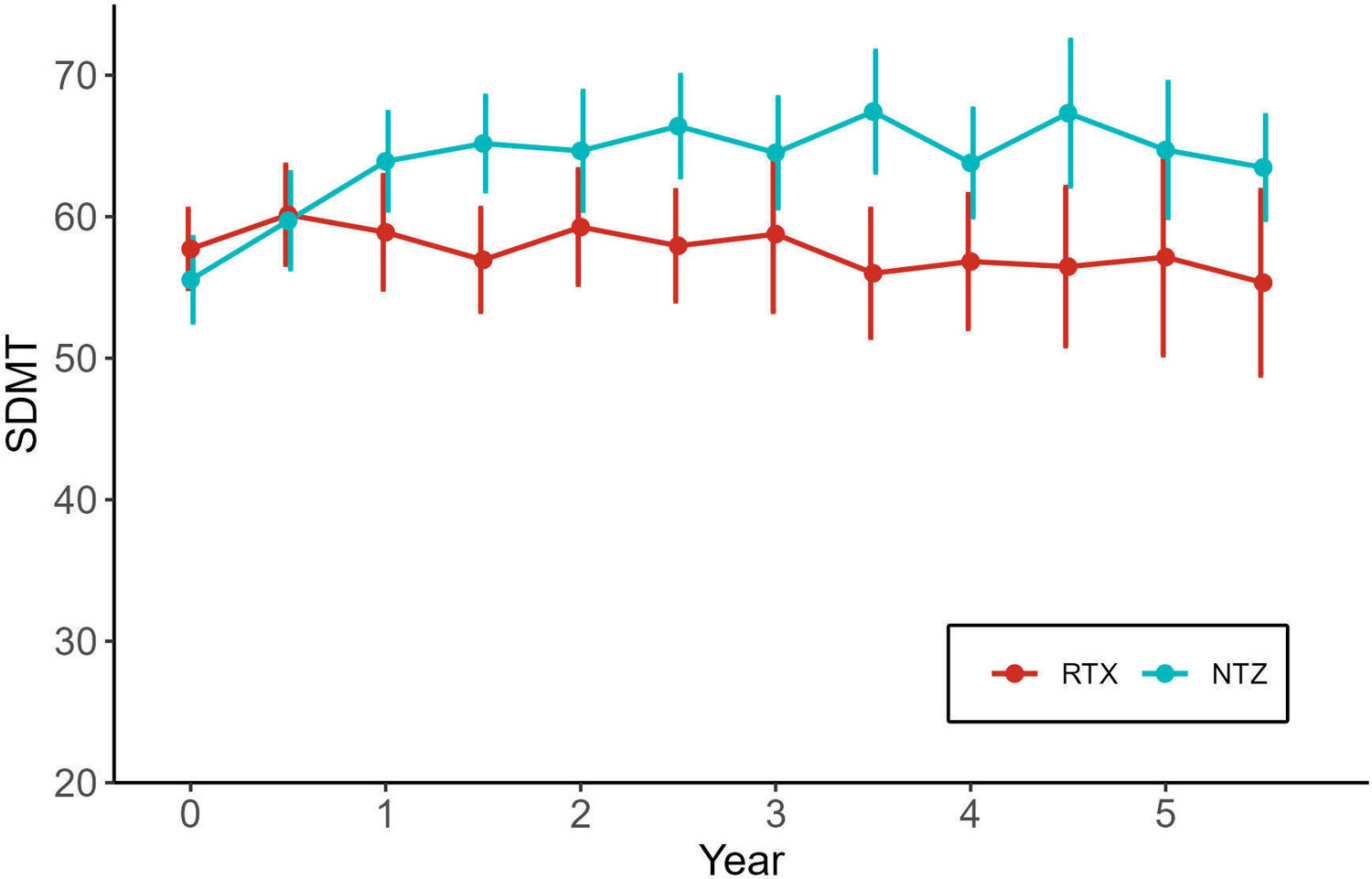
Depicts SDMT improvement on NTZ and RTX over the first five years from treatment initiation. The X-axis shows mean SDMT-scores with standard deviation. Mean values are displayed at the group level every six months. Abbreviations: SDMT, Symbol Digit Modalities Test; RTX, rituximab; NTZ, natalizumab.

Figure 5 displays the mean SDMT values at every six months from treatment initiation up to five years follow-up. The NTZ cohort displayed an improvement during the first year of treatment and then levelled off while the RTX cohort remained stable.

## Discussion

Fatigue is the most debilitating symptom to address in RRMS today as high-efficacy treatments often eliminate other aspects of disease activity. This study compared the high-efficacy drugs NTZ and RTX and found no significant difference in self-assessed fatigue by FSMC and health-related quality of life. In contrast, as secondary outcome measure, we found a significant difference between treatment groups in the attention domain, as measured by SDMT performance, with better results in the NTZ group.

The effects of NTZ and RTX on fatigue are not yet clear. Svenningsson et al. showed reduced fatigue levels in patients after initiation of NTZ.^20^ One study has evaluated the effect of RTX on MS-related fatigue with negative results.^15^ Previous comparative studies of fatigue have grouped DMTs into efficacy categories or by method of administration,^21,22^ making comparisons to our results difficult. In a recent study, Azoulai et al. evaluated fatigue in 300 patients with MS treated with high-efficacy drugs (NTZ, RTX, and ocrelizumab) in comparison with other DMTs.^21^ No significant differences between groups were seen suggesting that differences in fatigue between treatment options are likely to be small and in need of large study cohorts for detectability.

Notably, age stood out as the dominating confounder in both treatment groups, as shown in figures 3 and 4, with an increasing fatigue level at higher age spans for both drugs. Previous explorations of the correlation between age and fatigue yielded contradictory results. Lerdal et al. found strong correlations between the two, while Broch et al. and Razazian et al. did not.^23–25^ It is possible that the overall low level of physical impairment in our treatment groups made fatigue more apparent, and thus easier to detect the age association. Our study underscores the importance of adjustment for age in future comparative fatigue studies.

A larger proportion of participants in the RTX group were on fatigue medication, which may reflect that participants in the RTX group suffer from fatigue to a larger degree. We could only determine if a participant was prescribed fatigue medication, but not whether they used it daily, when needed or at all. Due to this uncertainty, we chose not to include fatigue medication as a confounder. We do not consider this to affect the self-reported fatigue in a major way as efficacy of these drugs has very little scientific support.^26^

We found significant improvement in information processing speed, in the attention domain of cognitive performance, as measured by SDMT over time in the NTZ group, a finding consistent with several previous studies.^27,28^ Manouchehrinia et al. found that patients treated with NTZ significantly improved in SDMT scores over the first six months of treatment compared to baseline, and that subjects on NTZ were more likely to have cognitive improvement than those on other monoclonal antibody therapies.^29^ The only previous study evaluating RTX by SDMT in patients with RRMS found a statistically significant improvement of 3.2 points at 24 months compared to baseline, in contrast to 8.9 in our NTZ cohort.^15^ Moreover, a change of ≤4 points was suggested to represent normal fluctuations in test performance by Strober et al. and a change of ≥8 points was proposed to indicate clinically meaningful improvement,^30^ which was reached in our study.

The main objection against a true improvement of SDMT performance is a potential practice effect of frequent testing. The majority of NTZ participants performed SDMT every 4–6 weeks while patients in the RTX cohort performed the test on average every 6 months. However, previous studies have shown that changing the version of the SDMT form at every testing occasion may eliminate the practice effect, and this was the general test regime at our study centres. Furthermore, a study by Perumal et al. tested SDMT at baseline and after 1 and 2 years of treatment, a test-retest interval that hardly would render a practice effect. Mean score improved 4.3 points which was statistically significant.^31–34^ However, in the end, we cannot rule out that participants developed improved strategies on answering the forms in our study.

In this study, there was a strong association between SDMT and the cognitive subscale of FSMC, as shown in Figure 4. Moreover, participants on NTZ had a higher SDMT score and a more favourable employment status than those on RTX. This is in accordance with previous studies that showed a linkage of SDMT performance with self-reported fatigue, work capability and socio-economic factors.^5,6,12,35^

### Strengths and limitations

Strengths of this study were the novel comparative approach with fatigue as a primary outcome between the two most used high-efficacy DMTs in a population-based sample, the prospective register-based collection of data based on geographical coverage, and the thorough statistical adjustment for multiple confounders. Limitations were inclusion bias as subjects with high fatigue burden might be more likely to participate, the limited number of participants posing a risk for underpowered calculations, and selection bias due to the substantial proportion of subjects that declined participation. The high number of declines (figure 1) were related to other investigations in the study protocol, i.e., a hesitance for lumbar puncture and a 7 T MRI with an extended protocol. Due to lack of data at treatment initiation, no longitudinal comparison FSMC values could be done. Lastly, but very importantly, there were no depression or anxiety measurements in our PROMs battery, conditions that strongly correlate with fatigue.

## Conclusions

We did not find any significant difference in self-assessed fatigue between two cohorts of patients treated with either natalizumab or rituximab in a population-based sample from Sweden. Our results support age as an important factor that should be considered when evaluating fatigue in MS. Patients treated with natalizumab displayed an improvement of information processing speed, an attention domain quality, not seen in patients treated with rituximab, a secondary study outcome. However, we cannot rule out that practice effect has affected the SDMT results. More in-depth studies regarding fatigue in relation to different DMTs in larger and controlled studies are warranted.

## Supplemental Material

sj-docx-1-mso-10.1177_20552173241252566 - Supplemental material for A comparative study of fatigue and processing speed in patients with multiple sclerosis treated with natalizumab or rituximab Supplemental material, sj-docx-1-mso-10.1177_20552173241252566 for A comparative study of fatigue and processing speed in patients with multiple sclerosis treated with natalizumab or rituximab by Johan Hellgren, Maria Compagno Strandberg, Kristina Källén and Anders Svenningsson in Multiple Sclerosis Journal – Experimental, Translational and Clinical
