# Supplementary material for: A comparative study of fatigue and processing speed in patients with multiple sclerosis treated with natalizumab or rituximab
Source: Mult Scler J Exp Transl Clin. 2024 May 26;10(2):20552173241252566. doi: 10.1177/20552173241252566 (PMC11131408; doi:10.1177/20552173241252566)
Supplement: sj-docx-1-mso-10.1177_20552173241252566 - Supplemental material for A comparative study of fatigue and processing speed in patients with multiple sclerosis treated with natalizumab or rituximab [file sj-docx-1-mso-10.1177_20552173241252566.docx]

# Full models

### Group comparison FSMC

#### FSMC continuous values

Linear regression was used for comparison between the two groups regarding the FSMC variables (cognitive/motor/total). The adjusted analysis includes the background variables deemed important to adjust for. In the sensitivity analysis, the patients who at the cross-section are on RTX, but were previously on NTZ, have been excluded.

|  |  | **Unadjusted Analysis** | | **Adjusted Analysis** | | **Sensitivity Analysis** | |
| --- | --- | --- | --- | --- | --- | --- | --- |
| **Outcome** | **Variable** | **Beta (95% CI)** | **P-value** | **Beta (95% CI)** | **P-value** | **Beta (95% CI)** | **P-value** |
| FSMC Cogn | RTX | 3.8 (-1.0 - 8.6) | 0.123 | -0.1 (-5.6 - 5.5) | 0.985 | 0.7 (-5.8 - 7.2) | 0.832 |
|  | Sex male |  |  | -6.2 (-12.4 - 0.0) | 0.051 | -3.6 (-10.8 - 3.7) | 0.331 |
|  | Age |  |  | 0.5 (0.2 - 0.8) | 0.004 | 0.4 (0.0 - 0.7) | 0.048 |
|  | Treat dur at incl |  |  | -0.4 (-1.5 - 0.6) | 0.414 | -0.3 (-1.5 - 0.8) | 0.586 |
|  | Disease dur |  |  | -0.1 (-0.5 - 0.3) | 0.493 | -0.2 (-0.6 - 0.3) | 0.447 |
|  | EDSS |  |  | 1.6 (-0.1 - 3.4) | 0.070 | 1.9 (-0.2 - 4.0) | 0.073 |
|  | Lesions at treatment init (ref: <10) |  |  |  | 0.201 |  | 0.411 |
|  | 10-20 |  |  | -4.3 (-11.0 - 2.4) | 0.209 | -2.8 (-10.3 - 4.7) | 0.465 |
|  | >20 |  |  | -5.2 (-10.9 - 0.5) | 0.076 | -4.2 (-10.4 - 2.0) | 0.184 |
| FSMC Motor | RTX | 4.6 (-0.2 - 9.4) | 0.058 | 0.5 (-4.8 - 5.8) | 0.857 | 0.8 (-5.3 - 6.9) | 0.793 |
|  | Sex male |  |  | -6.0 (-11.9 - 0.0) | 0.051 | -3.1 (-9.9 - 3.7) | 0.37 |
|  | Age |  |  | 0.5 (0.1 - 0.8) | 0.005 | 0.3 (-0.0 - 0.7) | 0.054 |
|  | Treat dur at incl |  |  | -0.4 (-1.5 - 0.6) | 0.398 | -0.4 (-1.5 - 0.7) | 0.446 |
|  | Disease dur |  |  | -0.0 (-0.4 - 0.3) | 0.811 | -0.1 (-0.5 - 0.3) | 0.724 |
|  | EDSS |  |  | 2.4 (0.7 - 4.1) | 0.006 | 3.0 (1.1 - 5.0) | 0.003 |
|  | Lesions at treatment init (ref: <10) |  |  |  | 0.401 |  | 0.718 |
|  | 10-20 |  |  | -4.0 (-10.5 - 2.5) | 0.224 | -2.1 (-9.1 - 5.0) | 0.562 |
|  | >20 |  |  | -3.4 (-8.9 - 2.1) | 0.227 | -2.4 (-8.2 - 3.5) | 0.424 |
| FSMC Total | RTX | 8.4 (-1.0 - 17.8) | 0.080 | 0.4 (-10.1 - 11.0) | 0.936 | 1.5 (-10.8 - 13.8) | 0.809 |
|  | Sex male |  |  | -12.1 (-24.1 - -0.2) | 0.046 | -6.6 (-20.4 - 7.1) | 0.338 |
|  | Age |  |  | 1.0 (0.3 - 1.6) | 0.004 | 0.7 (0.0 - 1.4) | 0.046 |
|  | Treat dur at incl |  |  | -0.9 (-3.0 - 1.2) | 0.396 | -0.7 (-2.9 - 1.5) | 0.506 |
|  | Disease dur |  |  | -0.2 (-0.9 - 0.6) | 0.634 | -0.2 (-1.0 - 0.6) | 0.564 |
|  | EDSS |  |  | 4.0 (0.6 - 7.4) | 0.021 | 4.9 (1.0 - 8.9) | 0.015 |
|  | Lesions at treatment init (ref: <10) |  |  |  | 0.284 |  | 0.544 |
|  | 10-20 |  |  | -8.3 (-21.3 - 4.7) | 0.207 | -4.8 (-19.1 - 9.4) | 0.501 |
|  | >20 |  |  | -8.6 (-19.6 - 2.5) | 0.126 | -6.5 (-18.3 - 5.2) | 0.272 |

### Group comparison SDMT

Linear regression was used for comparison between the two groups regarding SDMT. The adjusted analysis includes the background variables deemed important to adjust for. In the sensitivity analysis, the patients who at the cross-section are on RTX, but were previously on NTZ, have been excluded.

#### Table 1 Group comparison at cross section

| **Outcome** | **Variable** | **Unadjusted Analysis** | | **Adjusted Analysis** | | **Sensitivity Analysis** | |
| --- | --- | --- | --- | --- | --- | --- | --- |
|  |  | **Beta (95% CI)** | **P-value** | **Beta (95% CI)** | **P-value** | **Beta (95% CI)** | **P-value** |
| SDMT Cross-section | RTX | -8.5 (-12.9 - -4.0) | <0.001 | -8.2 (-13.5 - -2.8) | 0.003 | -7.4 (-13.1 - -1.7) | 0.012 |
|  | Sex Male |  |  | 1.5 (-4.0 - 7.1) | 0.585 | -0.5 (-6.4 - 5.5) | 0.876 |
|  | Age |  |  | -0.1 (-0.4 - 0.2) | 0.693 | -0.1 (-0.4 - 0.2) | 0.489 |
|  | Treat dur at incl |  |  | 0.0 (-1.0 - 1.0) | 0.970 | -0.0 (-1.1 - 1.0) | 0.925 |
|  | Disease dur |  |  | -0.1 (-0.4 - 0.2) | 0.610 | -0.1 (-0.5 - 0.2) | 0.432 |
|  | EDSS |  |  | -2.5 (-4.0 - -0.9) | 0.002 | -2.4 (-4.0 - -0.8) | 0.005 |
|  | Education |  |  | -3.1 (-8.1 - 2.0) | 0.234 | -3.1 (-8.6 - 2.4) | 0.264 |
|  | Lesions at treatment init (ref: <10) |  |  |  | 0.034 |  | 0.078 |
|  | 10-20 |  |  | 2.7 (-3.6 - 8.9) | 0.400 | 2.9 (-3.6 - 9.4) | 0.383 |
|  | >20 |  |  | -3.8 (-9.3 - 1.6) | 0.168 | -3.3 (-8.8 - 2.3) | 0.248 |

#### Table 2 Group comparison from treatment initiation to cross-section

| **Outcome** | **Variable** | **Unadjusted Analysis** | | **Adjusted Analysis** | | **Sensitivity Analysis** | |
| --- | --- | --- | --- | --- | --- | --- | --- |
|  |  | **Beta (95% CI)** | **P-value** | **Beta (95% CI)** | **P-value** | **Beta (95% CI)** | **P-value** |
| SDMT difference: cross-section – treatment initiation | RTX | -9.8 (-13.1 - -6.6) | <0.001 | -6.4 (-10.4 - -2.3) | 0.002 | -4.8 (-9.8 - 0.1) | 0.057 |
|  | Sex Male |  |  | -2.4 (-6.6 - 1.8) | 0.262 | -1.7 (-6.9 - 3.4) | 0.511 |
|  | Age |  |  | 0.0 (-0.2 - 0.3) | 0.677 | 0.0 (-0.2 - 0.3) | 0.887 |
|  | Treat dur at incl |  |  | 1.1 (0.3 - 1.8) | 0.005 | 1.1 (0.2 - 2.0) | 0.013 |
|  | Disease dur |  |  | -0.2 (-0.4 - 0.1) | 0.139 | -0.2 (-0.5 - 0.1) | 0.123 |
|  | EDSS |  |  | -0.9 (-2.1 - 0.3) | 0.141 | -0.9 (-2.3 - 0.6) | 0.230 |
|  | Education |  |  | -1.6 (-5.5 - 2.3) | 0.416 | -0.9 (-5.6 - 3.9) | 0.716 |
|  | Lesions at treatment init (ref: <10) |  |  |  | 0.530 |  | 0.264 |
|  | 10-20 |  |  | 2.7 (-2.0 - 7.5) | 0.261 | 4.4 (-1.2 - 10.1) | 0.125 |
|  | >20 |  |  | 1.6 (-2.6 - 5.7) | 0.459 | 3.3 (-1.5 - 8.2) | 0.174 |
